# Catastrophic thrombotic events with partial bilateral amputation of legs and fingers in a 12-year-old girl with COVID-19 in Brazil: case report

**DOI:** 10.1590/1677-5449.202301752

**Published:** 2024-08-09

**Authors:** Giselly Rosa Modesto Pereira, André Ricardo Araújo da Silva, Claudia Escórcio Gurgel do Amaral Pitanga, Jocemir Ronaldo Lugon

**Affiliations:** 1 Universidade Federal Fluminense – UFF, Niterói, RJ, Brasil.; 2 Associação Fluminense de Reabilitação, Núcleo de Estudos, Projetos e Pesquisas, Niterói, RJ, Brasil.

**Keywords:** case report, SARS-CoV-2, COVID-19, children, thrombosis, amputation, relato de caso, SARS-CoV-2, COVID-19, criança, trombose, amputação

## Abstract

The first case of COVID-19 was detected in Dec 2019, in China. The disease shortly evolved into a pandemic and imposed an unparalleled health and social burden on mankind. Severe forms of COVID-19 mainly affect adults, especially the elderly and those with comorbidities. We report a severe case of COVID-19 in a previously healthy 12-year-old female who was admitted to the emergency room on May 26, 2020, with fever, abdominal pain, vomiting, and diarrhea. During the hospital stay, she tested positive for SARS-CoV-2 and developed multiple organ failure and catastrophic thrombotic events resulting in bilateral amputation of legs and fingers. She was discharged from the hospital for outpatient follow-up after 107 days. By the time this report was written, the patient was undergoing prosthesis prescription and training and regaining her independence to walk.

## Resumo

O primeiro caso de covid-19 foi detectado em dezembro de 2019, na China. A doença rapidamente evoluiu para uma pandemia e trouxe um fardo social e de saúde sem paralelo para a humanidade. As formas graves de covid-19 afetam principalmente adultos, especialmente idosos com comorbidades. Relatamos um caso grave de covid-19 em uma paciente de 12 anos de idade, previamente saudável, que deu entrada no pronto-socorro em 26/05/2020 com febre, dores abdominais, vômitos e diarreia. Durante a investigação, apresentou teste positivo para SARS-CoV-2 e evoluiu com falência múltipla de órgãos e eventos trombóticos catastróficos, resultando em amputação bilateral de pernas e quirodáctilos. Ela recebeu alta hospitalar para acompanhamento ambulatorial após 107 dias. No momento da redação deste relato, a paciente estava sendo submetida a protetização e recuperando marcha independente.

## INTRODUCTION

COVID-19, an infection caused by the SARS-CoV-2 first reported in China at the end of 2019, imposed an unparalleled health and social burden on humanity.^1^ The first case in Brazil of the SARS-CoV-2 infection was reported on February 26, 2020. As of April 2023, there were about 37,000,000 cases in Brazil, and the country ranked 5th in the world in the number of cases, 96th in cases per million population, and 20th in deaths per million population.^2^

Severe forms of the disease are known to mainly affect adults, especially the elderly and those with comorbidities such as diabetes mellitus and obesity.^3^ Most children with COVID-19 have mild forms of the disease.^4^ We report a severe case of COVID-19 in a child occurring during the second month of the pandemic in Brazil that progressed with catastrophic thrombotic events, requiring bilateral partial amputation of the legs and fingers.

This case report was approved by the research ethics committee at the Medical School of the Universidade Federal Fluminense, under approval number 4.100.232. Informed consent was obtained from the patient’s parents.

## CASE REPORT

A 12-year-old female Caucasian, 50 Kg, was admitted on May 26, 2020, with suspected Covid-19. She had fever, abdominal pain, vomiting and diarrhea with onset 4 days earlier, and a skin rash that appeared on the day of admission. Her vaccinations were up to date with the regular Brazilian vaccination schedule and she had previously been admitted to hospital for asthma, but was not regularly using any medication. Initial laboratory test results were as follows: type A+ blood, hemoglobin 11g/dL, leukocytes 6,100/mm^3^, platelets 154,000/mm^3^, BUN 4.9 mg/dL, creatinine 0.59mg/dL, Na 134mEq/L, K 4mEq/L, Cl 102mEq/L, ionized Ca 1.06 mmol/L, Mg 1.74mg/dL, phosphate 3.43mg/dL, AST 28.9 U/L; ALT 24.1 U/L; GGT 15 U/L; total bilirubin 0.28mg/dL; alkaline phosphatase 127 U/L; LDH 433 U/L; PT/INR 14.5 sec/1.27; aPTT 33.5 sec; D-dimer 1,847 ng/mL; and C-Reactive protein 9.96mg/dL. Her O_2_ saturation was 98% on room air. One day after admission, she had to be admitted to the intensive care unit (ICU) because of tachycardia, suspected myocarditis on an echocardiogram, and pneumonia (bilateral and peripheral ground-glass pulmonary opacities and consolidation on a chest computed tomography). Troponin levels were within the reference range. She was started on enoxaparin (1mg/kg q.d.), cefepime, and azithromycin. She developed a diffuse macular rash affecting the lower and upper limbs. On the 2nd day, she had arterial hypotension, psychomotor agitation, severe tachypnea, and great difficulty breathing on nasal oxygen at 10 L/min – saturation was 93%. She underwent orotracheal intubation and was placed on mechanical ventilation. Kidney replacement therapy (KRT) was started on the 3rd day and the same day an oropharyngeal swab tested positive for SARS-Cov-2 by RT-PCR. She underwent a platelet transfusion on the 4th day because of marked thrombocytopenia (platelet count 25.000/mm^3^), which was repeated on the following day because of bleeding at the puncture sites and nose. On the 6th day, her lower and upper extremities became cyanotic. On the 8th day, a vascular surgeon’s opinion was requested. Serum D-dimer was 638,250 ng/ml. She was switched from enoxaparin to intravenous unfractionated heparin 10UI/kg/h, which was maintained along with the ICU stay.

**Figure 1:**
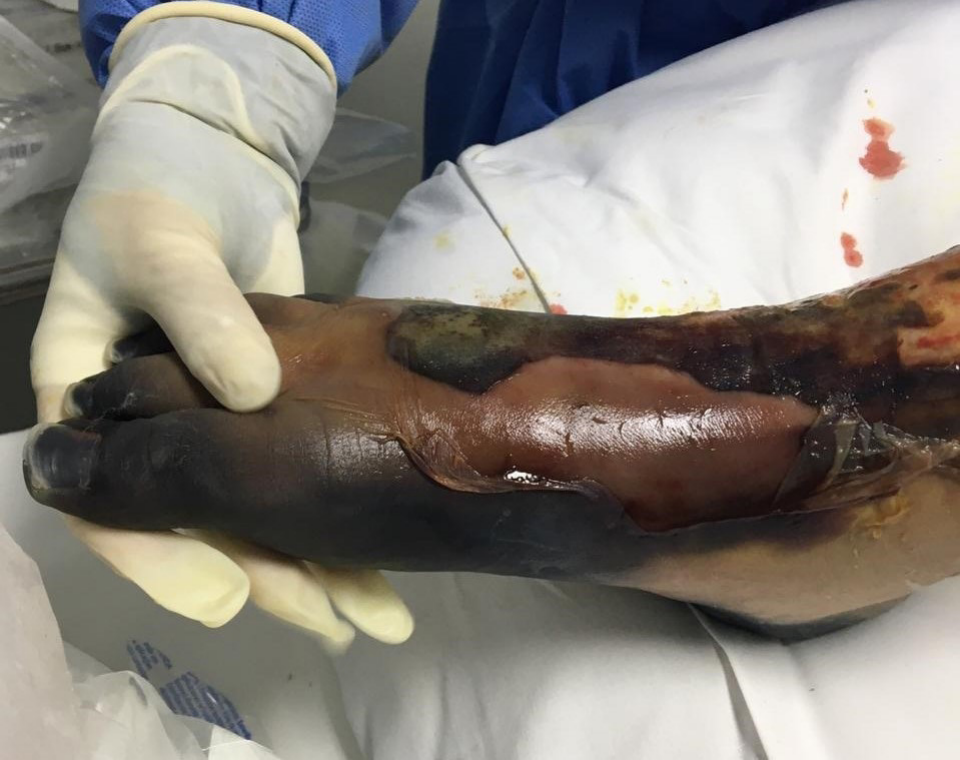
Severe ischemia of the right foot – lesions were bilateral.

**Figure 2:**
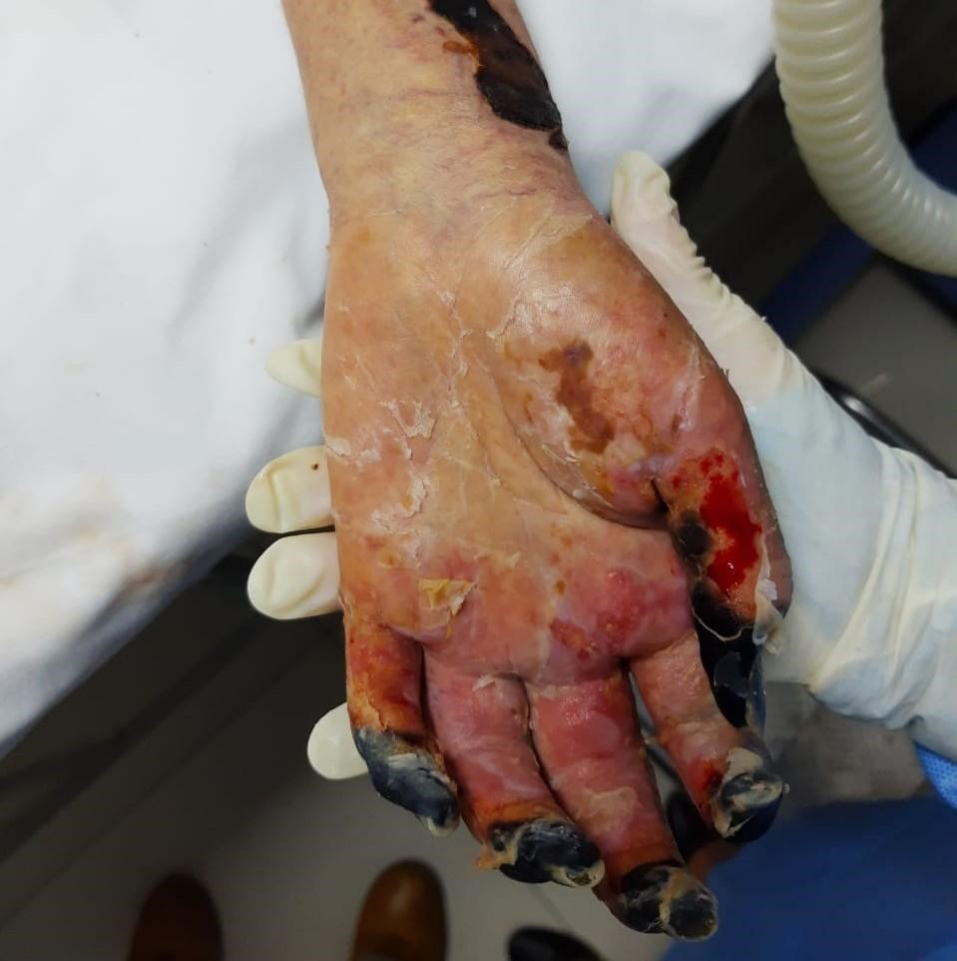
Severe ischemia of the distal extremities of the left fingers – lesions were bilateral.

She underwent her first blood transfusion on the 11th day. Pulmonary hemorrhage and pleural effusion were detected on the 15th day. The family was informed that surgical resection of the feet and digital extremities was necessary because of serious limb ischemia (Figures 1 and 2).

She underwent tracheostomy on the 23rd day. On the 27th day, daily hyperbaric chamber sessions were initiated and were maintained up to the 34th day. On that day, she was diagnosed with bacterial conjunctivitis in the right eye and topical dexamethasone and tobramycin were started. On the 37th day, amputation of the lower limbs was performed below the patella because of worsening of the ischemia. On the 42nd day, she exhibited features of phalangeal necrosis of both hands and systemic infection by pseudomonas. The mother and the patient received counselling from the mental health care team regarding the need for surgery before amputation of the distal phalanges of both hands was performed. She developed pneumonia from a nosocomial *Acinetobacter* sp. infection and amikacin and piperacillin/tazobactam were started. A right corneal perforation was initially managed with a contact lens and topical antibiotics. Her progressively worsening nutritional status prompted an endoscopic gastrostomy. A right corneal transplant was performed on the 101st day. She improved gradually and was discharged after 107 days in hospital, weighing 40 kg, to start home care treatment without anticoagulation.

**Figure 3:**
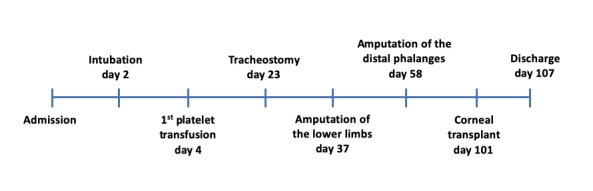
Timeline of the key points during hospitalization.

The key events during the hospital stay are illustrated in Figure 3.

The case report was written following the CARE guidelines.^5^

## DISCUSSION

We report a severe COVID-19 case in a 12-year-old female, which occurred early in the pandemic when knowledge about management of the disorder was very incipient and no vaccine for SARS_CoV-2 was available. The disease rapidly involved the gastrointestinal system, heart, lungs, and kidneys in association with catastrophic thrombotic events that caused significant sequelae before hospital discharge.

Involvement of the heart was manifest with tachycardia and motility disturbances shown by an echocardiogram, prompting admission to the ICU. Acute myocarditis was initially thought of as a rare manifestation of COVID-19, but is now recognized as relatively frequent. The exact prevalence of acute myocarditis in COVID-19 remains unknown. In a large literature review of autopsy studies, the prevalence rate varied between 1.4 and 7.2%.^6^ In clinical settings, it has been reported in 20-36% of cases with a severe form of the disease depending on the diagnostic criteria employed.^7^ Close to 80% of affected patients have elevated troponin, which was not found in our patient. Nonetheless, the heart involvement was not a major determinant of the course of the disease in the present case.

This patient developed an acute respiratory syndrome, very common in severe COVID-19 cases.^8^ Pulmonary involvement can be very distressing and require ICU admission – not rarely, it can be a major determinant of prognosis and responsible for a fatal outcome. In a meta-analysis comprising 28 studies, the ICU admission rate of hospitalized cases was 21%, 67% of which needed mechanical ventilation.^9^ Severe forms of lung disease are uncommon in children and the role of the patient’s previous diagnosis of asthma as a risk factor is still debatable.^4^ In the case reported herein, the patient was successfully weaned, remaining on spontaneous breathing until discharge, despite the prolonged period of mechanical ventilation during which she had multiple respiratory infections.

Acute renal injury (ARI) occurred very early during her ICU stay, demanding KRT on the third day. Overall, the incidence of ARI in people with COVID-19 has ranged from 5% to 29% with great variation between centers, possibly due to differences in population demographics and comorbidities.^10^ Its pathophysiology is still controversial.^11^ Kidney function slowly recovered allowing discontinuation of KRT after 30 days. By the time of hospital discharge, our patient’s creatinine serum levels were well within the reference range (BUN 6.8mg/dL and Cr 0.48 mg/dL).

The complications that made this case unique and motivated this report were those related to the coagulation system. Arterial thromboses have been reported in adults with severe forms of COVID-19,^12^ but in children, we only found one report from India, in which amputation of the right limb was necessary in a neonate.^13^ Our patient exhibited marked thrombocytopenia early in the course of the disease in association with thrombotic and bleeding events. Despite the efforts to treat her condition, she had poor progress, resulting in bilateral infrapatellar amputation of the lower limbs and partial amputation of the fingers of both hands. Thrombocytopenia is a common finding in COVID-19, present in 5–42% depending on disease severity.^14^ Its association with thrombotic events such as seen in the present case seems to signal a particularly dismal prognosis.^15^ Many mechanisms have been proposed for the thrombocytopenia in COVID-19: increased platelet aggregation and microthrombi formation, increased extraction of hyperactive platelets by macrophages, and direct or indirect hematopoiesis disturbances.^16^ However, the early onset of thrombocytopenia and its association with catastrophic thrombotic events lead us to think that a massive increase in platelet aggregation may have been the predominant phenomenon in our patient.

It remains to be clarified whether the increased aggregation is induced by direct platelet activation by the virus or the associated cytokine storm, or is secondary to the endothelium damage provoked by the SARS-CoV-2 infection.^16^ Unfortunately, physicians are still faced with a dilemma regarding the best therapeutic choice in this scenario.^17^ Similar to the strategy adopted for our patient, a recent guideline for anticoagulation in severe forms of COVID-19 with thrombosis recommends use of higher-intensity therapeutic anticoagulation with low molecular weight heparin or unfractionated heparin.^18^ However, the guideline acknowledges the lack of high-quality randomized controlled trials comparing different intensities of anticoagulation for patients with COVID-19-related critical illness.

Finally, infective conjunctivitis, corneal perforation, and endophthalmitis were managed with antibiotics and cornea transplantation, which failed. Although there are reports of eye lesions related to SAR-CoV-2 infection, the eye complications in this case more probably reflect exposure keratopathy as the initial lesion, which is common in patients undergoing long-term intensive care treatment under neuromuscular block and sedation.^19^

**Figure 4:**
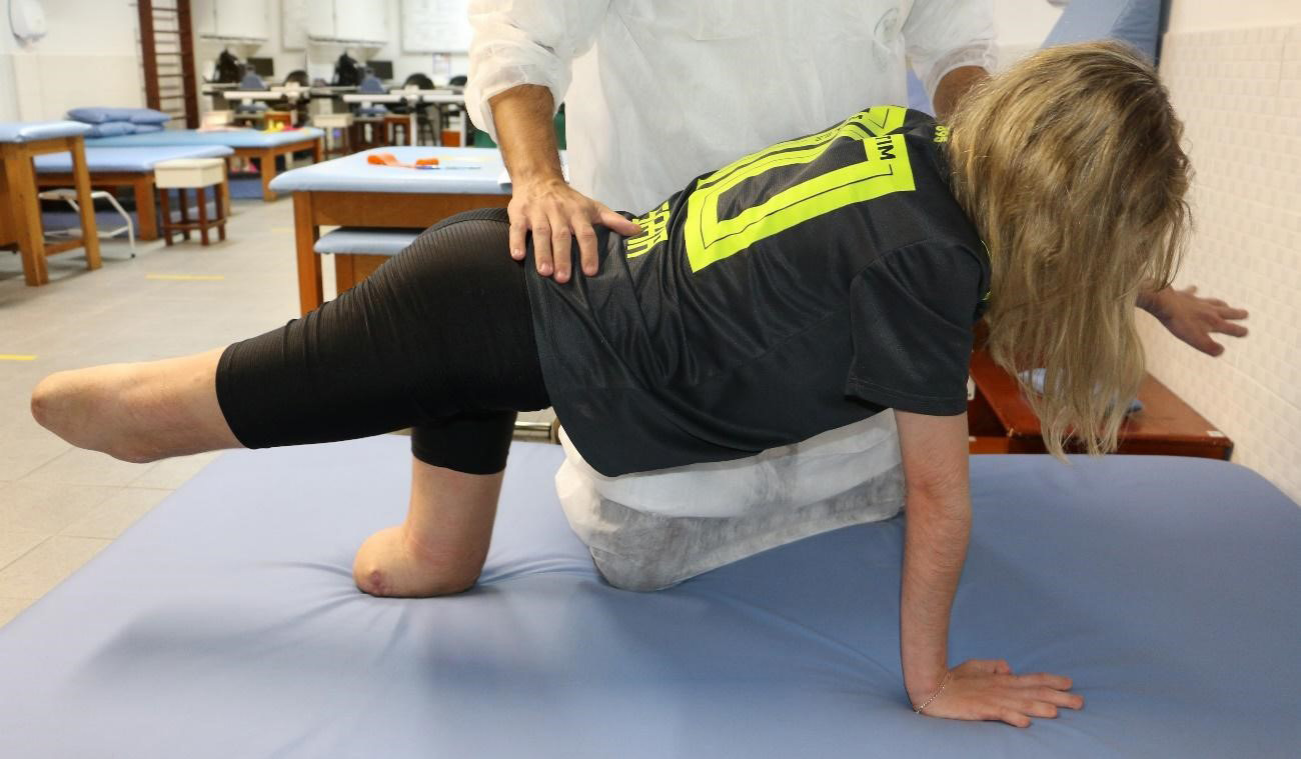
Rehabilitation session 6 months after hospital discharge.

At the time the report was written, the patient was doing well, with intact cognition, regular oral feeding, spontaneous breathing, and normal renal function. A second corneal graft was performed later after her hospital discharge, partially recovering sight in her right eye. She was undergoing rehabilitation with prosthesis prescription and training and regaining her independence to walk (Figure 4).

In conclusion, the present case report demonstrates that COVID-19 in children can have a severe course and can result in significant sequelae. Early use of drugs that effectively block viral multiplication such as remdesivir, for instance, is expected to be an important complement to the supportive treatment of severe cases of COVID-19 in the future.
